# Exploring the Prevalence and Biological Features of Astigmatism in Primary School Children in Zibo City, China

**DOI:** 10.1155/joph/8066266

**Published:** 2025-11-19

**Authors:** Chuanxian Qiu, Yunfei Han, Lihua Zhang, Huijuan Xu, Linlin Xu

**Affiliations:** ^1^Department of Ophthalmology, Linzi Maternal and Child Care Service Centre (Qidu Hospital), No 123 Dashun Road, Linzi 255400, Shandong, China; ^2^Department of Refractive Surgery, Binzhou Hubin Aier Eye Hospital, No 1017 Huanghe 12 Road, Binzhou 256600, Shandong, China; ^3^Department of Otorhinolaryngology and Head and Neck Surgery, Zibo Central Hospital, No 10 Shanghai Road, Zhangdian District, Zibo 255000, Shandong, China; ^4^Department of Ophthalmology, Zibo Central Hospital, No 10 Shanghai Road, Zhangdian District, Zibo 255000, Shandong, China

**Keywords:** astigmatism, children, ocular biometrics, prevalence

## Abstract

**Objective:**

To evaluate the prevalence of astigmatism and its association with ocular biometrics in primary schoolchildren in Zibo City, China.

**Methods:**

A cross-sectional study was conducted among 25,140 children aged 7–13 years from 19 primary schools in Linzi District, Zibo City, China, between April and May 2024. Refractive status was assessed via autorefractometry under natural pupil conditions, and astigmatism prevalence was determined using right-eye data. The study evaluated astigmatism severity (mild, moderate, or high), axis orientation (with-the-rule, against-the-rule, or oblique), visual acuity, and correlations with spherical refraction. A nested case–control analysis was performed to compare ocular biometric parameters, including axial length (AL), central corneal thickness (CCT), corneal diameter (white-to-white, WTW), and lens thickness (LT), among groups stratified by astigmatism severity.

**Results:**

The overall prevalence of astigmatism was 46.21% (95% CI: 45.6%–46.8%), increasing with advancing age and school grade. Astigmatism severity distribution was as follows: mild (37.16%), moderate (6.81%), and high (2.24%) among all participants. Among the 11,616 children with astigmatism, 62.86% had with-the-rule astigmatism, 27.48% exhibited against-the-rule astigmatism, and 9.66% presented oblique astigmatism. Visual acuity exhibited a significant decline with increasing astigmatism severity (*p* < 0.001). Higher astigmatism severity was inversely associated with myopia prevalence (*p*=0.04). Ocular biometric parameters, including AL, CCT, WTW, and LT, showed significant differences across severity groups (*p* < 0.05).

**Conclusion:**

Astigmatism is highly prevalent among primary school children in Linzi District, with significant associations between severity, ocular biometrics, and visual outcomes. These findings emphasize the need for early screening and tailored management strategies for childhood astigmatism.

## 1. Introduction

Astigmatism is a refractive error caused by irregular curvature of the cornea or lens, creating uneven light focusing across perpendicular meridians and causing blurred vision at all distances [1]. Unlike spherical errors (myopia/hyperopia), it can occur independently or coexist with them. As a common refractive error, astigmatism significantly affects visual quality and may hinder the normal development of vision, especially in children [2, 3].

There are significant differences in the prevalence of astigmatism among children worldwide due to racial and regional variations, with Asians, African Americans, and Hispanic individuals being more prone to astigmatism than non-Hispanic Whites [4, 5]. The prevalence of astigmatism among children in China is 16.5%, higher than the Asian average of 14.9% [6]. The research results on the prevalence of astigmatism among children in China vary due to differences in region, population, and criteria for defining astigmatism [7–9]. Overall, the prevalence is higher in the south than in the north, and higher in urban than in rural areas [6]. And during the COVID-19 pandemic, this rate was significantly higher than before the pandemic, which may be related to the reduction in outdoor activities, increased online classes, and academic workload for children after the pandemic [10]. With the increasing burden of eye conditions globally, understanding the prevalence and associated factors of astigmatism is crucial for the allocation of health resources and the implementation of preventive measures.

Refractive errors, particularly myopia, and their relationships with various ocular biometric parameters have long been a topic of interest for researchers [11, 12]. Ocular biometry involves measurements such as the axial length (AL), corneal curvature, and lens thickness (LT), which are crucial in determining the eye's refractive status [13]. However, studies investigating the association between astigmatism and ocular biometric parameters, especially in pediatric populations, remain extremely rare. We believe that exploring these correlations is critical for gaining deeper insights into the etiologies of astigmatism and developing potential interventions.

This study aimed to investigate the prevalence of astigmatism among primary school students in Linzi District, Zibo City, China, and to explore the association between astigmatism and ocular biometric parameters. By providing critical epidemiological evidence regarding refractive errors in pediatric populations, this research seeks to enhance our understanding of the pathophysiological mechanisms underlying astigmatism development and its ocular correlates. The findings are expected to contribute valuable insights for optimizing pediatric eye care strategies and informing public health initiatives targeting refractive error prevention in school-aged children.

## 2. Methods

### 2.1. Study Design and Participants

This cross-sectional study with a nested case–control analysis was conducted in the Linzi District of Zibo City, Shandong Province, China, from April to May 2024. The investigation employed a school-based cluster sampling design, with primary schools as the primary sampling units. A total of 19 schools were randomly selected from a total of 36 eligible primary schools in the district, encompassing a study population of 25,140 children aged 7–13 years. Right-eye data were used for the analysis to ensure standardized measurement, with justification provided in the methodology section. The prevalence of astigmatism was assessed using standardized ocular examinations. Participants were excluded if they had active ocular pathologies (e.g., corneal opacity, cataracts, glaucoma, and retinal lesions) or a history of ocular trauma or surgery.

Case–Control Sampling and Group Stratification: From the screened population, participants with astigmatism (cylindrical power ≤ −0.50 diopters [D] in the right eye) were stratified into three severity groups based on refractive error: mild, moderate, and high astigmatism. A stratified random sampling approach was applied to optimize statistical power while accounting for resource constraints. Participants were first stratified by astigmatism severity mild, moderate, severe, age 7–9 years versus 10–12 years, and sex. Within each stratum, participants were randomly selected using a computer-generated random number sequence. Sample sizes were determined via power analysis G∗Power 3.1 assuming an effect size of 0.5 moderate difference in AL, *α* = 0.05, and 80% power, requiring a minimum of 50–70 participants per group. The final allocation mild: *n* = 96, moderate: *n* = 80, and severe: *n* = 64 ensured sufficient representation of severe cases while maintaining statistical rigor. All measurements were performed by technicians blinded to participants' group assignments.

### 2.2. Examinations

All ocular assessments were performed by three trained and certified ophthalmologists, who underwent standardized training to ensure consistency in measurements. Examinations followed a structured protocol to minimize interobserver variability. Noncycloplegic Refraction: Refractive error was measured using an autorefractor (Cannon RF10, Tokyo, Japan). Participants were seated at a standard distance, and noncycloplegic autorefraction was performed on the right eye only. Three consecutive measurements were taken in SET mode, and the mean value was recorded to ensure accuracy. Visual acuity (VA) was measured monocularly using a Snellen chart with E optotypes (GB 11533-2011) at a 5-m distance under standardized illumination (500 lux). Children first identified optotypes without refractive correction, followed by measurement of corrected VA using their habitual spectacles (if available). Uncorrected VA was classified as abnormal if the LogMAR score exceeded 0.1 (equivalent to 20/25 Snellen), aligning with WHO guidelines for pediatric vision screening. Ocular Biometric Measurements: Corneal curvature (K), AL, and other parameters were obtained using the IOL Master 700 (ZEISS, Germany). The following parameters were systematically recorded for each eye: AL, CCT, WTW, LT, the ratio of axis length and keratometry (A/K ratio), and K value.

### 2.3. Definition

Myopia occurs when the eye's refractive power exceeds its AL requirements, causing light to focus in front of the retina when relaxed. Myopia was defined as spherical equivalent (SE) ≤ −0.50 D, mild myopia as −3.00 D ≤ SE ≤  −0.50 D, moderate myopia as −6.00 D ≤ SE <  −3.00 D, and high astigmatism as *C* <  −6.00 D [1].

Hyperopia occurs when the eye's refractive power is insufficient relative to its AL, causing light to focus behind the retina when relaxed. Hyperopia was defined as SE > 0.00 D, mild hyperopia as 0.00 D < SE ≤ +3.00 D, moderate myopia as +3.00 D < SE ≤ +5.00 D, and high astigmatism as SE > +5.00 D.

Astigmatism occurs when incoming parallel light forms two focal lines due to uneven refraction in the eye, creating a minimal circle of confusion between them.

Astigmatism was categorized based on cylinder power (C): mild astigmatism was defined as −1.00 D < *C* ≤  −0.5 D, moderate astigmatism as −2.00D < *C* ≤  −1.00 D, and high astigmatism as *C* ≤  −2.00 D.

The astigmatism was categorized based on different axis as follows: with-the-rule astigmatism (maximum refraction of the main meridian in 180° ± 30°), against-the-rule astigmatism (maximum refraction of the main meridian at 90° ± 30°), and oblique astigmatism (all other cases).

### 2.4. Ethical Approval

This study was approved by the Institutional Review Board of Linzi Maternal and Child Care Service Center (Qidu Hospital; Approval No. 2023-002) and complied with the Declaration of Helsinki and ICH Good Clinical Practice guidelines. Written informed consent was obtained from all parents/guardians, with additional verbal assent from children aged ≥ 12 years. The study involved only noninvasive, low-risk ocular examinations (e.g., autorefraction). Participants could withdraw anytime without penalty, and all data were anonymized and securely stored. The research aimed to enhance understanding of pediatric astigmatism, potentially benefiting public health strategies.

### 2.5. Statistical Analysis

Data analysis was performed using IBM SPSS Statistics Version 24.0 (IBM Corp., USA). Continuous variables were tested for normality using the Shapiro–Wilk or Kolmogorov–Smirnov test and presented as mean ± SD (normally distributed) or median (interquartile range) otherwise. Group comparisons for normally distributed data used one-way ANOVA with post hoc Tukey's or Bonferroni correction; non-normal data were analyzed via Kruskal–Wallis tests. Categorical variables were summarized as frequencies (%) and compared using Pearson's chi-square test; Fisher's exact test was applied for small sample sizes < 5. A two-tailed *p* < 0.05 indicated significance, with Bonferroni correction applied for multiple comparisons to control the family-wise error rate.

## 3. Results

### 3.1. Characteristics of the Study Population

As shown in Table 1, the cross-sectional survey was conducted during the spring semester of 2024 and enrolled a total of 25,140 students, comprising 14,014 boys and 11,126 girls (male-to-female ratio: 1.26:1). The cohort had a mean age of 9.17 ± 1.52 years. Participants were stratified by grade level: students aged 7–10 years (corresponding to lower primary Grades 1–4) accounted for 19,320 individuals (76.8% of the cohort), while those aged 11–13 years (upper primary grades 5–6) represented 5820 students (23.2%).

We conducted a comparative analysis of VA, spherical refraction (sphere power), cylindrical refraction (cylinder power), and astigmatism axis orientation between lower and upper primary school grades. As age and grade level advanced, we observed a significant decline in mean VA, accompanied by progressive increases in both spherical and cylindrical refractive errors (measured in diopters). Additionally, a statistically significant difference in astigmatism axis orientation was identified between the two groups (Table 2).

### 3.2. Prevalence of Astigmatism

Using the predefined criteria for astigmatism, our study identified 11,616 cases of astigmatism, yielding an overall prevalence of 46.21% (95% CI: 45.6%–46.8%). Stratified by gender, the prevalence was 56.13% among males and 43.87% among females. Astigmatism severity was further categorized as follows: mild astigmatism accounted for 37.16%, moderate astigmatism represented 6.81%, and high astigmatism constituted 2.24% (Table 1).

### 3.3. Distribution of Astigmatism Axis

**Figure 1:**
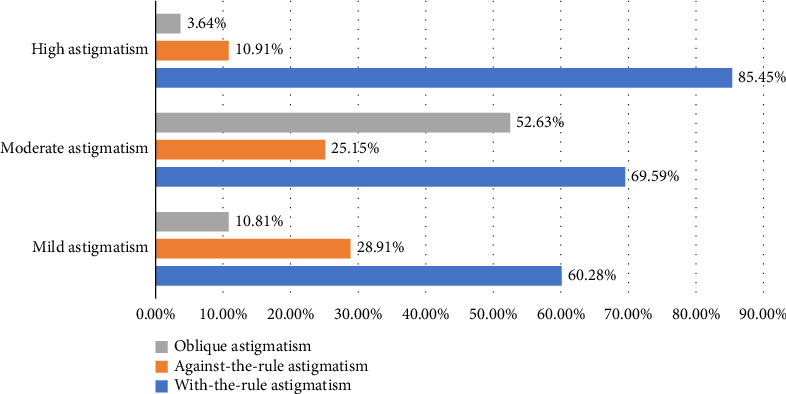
Axial variations in astigmatism across different degrees.

Among the 11,616 students with astigmatism, 7302 (62.86%) had with-the-rule astigmatism, 3192 (27.48%) had against-the-rule astigmatism, and 1122 (9.66%) had oblique astigmatism. Within the group of students with a cylinder power *C* of ≤ 0.5 D, the type of astigmatism varied according to the degree of astigmatism as illustrated in Figure 1.

### 3.4. Relationship Between Astigmatism and Spherical Refraction

As illustrated in Table 3, our analysis meticulously investigated the disparities in age, VA, and spherical refractive error among students categorized by different degrees of astigmatism. It is noteworthy that no significant variations in age were detected across the categorized groups. In contrast, a progressive deterioration in VA was observed with increasing degrees of astigmatism, a finding that reached statistical significance (*p* < 0.001). Moreover, the examination revealed discernible differences in myopia levels across the groups, especially when comparing students with mild to those with high astigmatism. This association demonstrated a clear pattern: As the degree of astigmatism escalated, a decline in myopia levels followed suit, indicating a significant correlation that commands attention (*p*=0.04).

### 3.5. Comparison of Biological Parameters of Eyes With Different Degrees of Astigmatism

Table 4 showcases the comparative analysis of ocular biometric parameters across three groups, revealing significant disparities in Axl, CCT, A/K, LT, and W-W values. Notably, the mild group demonstrated the longest Axl at 24.06 ± 0.888 mm, succeeded by the moderate group at 23.66 ± 1.376 mm, and the high group at 22.77 ± 1.189 mm (*p* < 0.001). In terms of CCT, the high group recorded the lowest measurements 528.97 ± 31.544 μm, as opposed to the moderate and mild groups, which measured 536.64 ± 26.815 μm and 540.29 ± 24.221 μm, respectively (*p*=0.03). The A/K ratio findings mirrored the Axl results, with the mild group leading at 3.06 ± 0.117, contrasting with the moderate group at 3.01 ± 0.177, and the high group at 2.98 ± 0.540 (*p*=0.01). Conversely, LT demonstrated an inverse relationship with astigmatism severity; the high group had the highest LT 3.50 ± 0.201 μm, followed by the moderate Group 3.40 ± 0.215 μm and then the mild Group 3.40 ± 0.189 μm (*p* < 0.001). Additionally, the difference of WTW among the three groups was found to be statistically significant, with the moderate astigmatism group presenting the greatest discrepancy at 12.30 ± 0.417 mm.

Other assessed parameters, such as age, gender, K1, and axis, did not exhibit significant variations among the groups (*p* > 0.05). Additionally, RA and CA in each group demonstrated consistency in both magnitude and axis orientation.

## 4. Discussion

Astigmatism is more prevalent in infants and young children than in adults [14, 15]. Recent studies highlight age- and region-specific variations in astigmatism prevalence among Chinese children. Wang et al. found 14.2% astigmatism prevalence in 4801 eastern Chinese schoolchildren (mean age 12.3 years) and 2.2% as high astigmatism [16]. This aligns with Zhang et al.'s 16.5% prevalence in Shanghai children aged 4–15 years [17]. However, Yang's study in Wuxi reported a much higher 36.0% prevalence in preschoolers (under six), suggesting age-related differences in susceptibility or measurement approaches [18]. A large-scale study in Xi'an involving 99,515 students aged 6–18 years reported an astonishingly high prevalence of astigmatism at 59.3%, which may be attributed to age-related factors, regional factors, urbanization, or broader diagnostic criteria [19]. The dominance of with-the-rule astigmatism in school-age groups highlights its demographic prevalence, while preschoolers' higher rates warrant further study into transient refractive changes or early risk factors.

This study revealed a high prevalence of astigmatism among primary school children in Linzi District, Zibo, being 47.33%, slightly higher than most other areas. This may be related to the relatively developed economy in Linzi District, the presence of many large enterprises, a higher level of education among parents, a greater emphasis on children's education, early learning among children, and a lack of outdoor activities. Additionally, differences in the instruments used for examination in various studies can also lead to differences in the prevalence rates [20]. Astigmatism in childhood mainly originates from the cornea and is related to the pressure exerted by the eyelids on the eyeball [21]. It manifests primarily as with-the-rule astigmatism, followed by oblique astigmatism, with against-the-rule astigmatism being the least common [8, 17, 22, 23]. In this study, the proportion of with-the-rule astigmatism in preschool children increases with the degree of astigmatism, reaching up to when astigmatism is ≥ 2 D. Furthermore, astigmatism often coexists with spherical refractive errors. The main types of astigmatism in preschool children are compound hyperopic astigmatism and mixed astigmatism, while simple astigmatism and compound myopic astigmatism are less common [17]. However, since the data collected in this study were the refractive errors measured without cycloplegic paralysis, no analysis was performed on the types of astigmatism.

It is well established that high astigmatism (≥ 2.00 D) invariably results in abnormal vision that is often below the normal level for their age group, making it highly prone to causing amblyopia, with vision decreasing by one line for every 0.25 D increment in astigmatism [24, 25]. This study demonstrates a statistically significant positive correlation between astigmatism severity and the prevalence of visual impairment among primary schoolchildren in Zibo City. When the degree of astigmatism is less than 1 D, vision is basically normal, while vision falls below normal levels when the degree of astigmatism is ≥ 1 D. This highlights the critical need for early referral of children with signs of low vision to an ophthalmology clinic to rule out potential eye diseases and refractive errors. Should refractive screening be available, a cylinder degree exceeding 1.25 D is proposed as the threshold for referring cases of abnormal astigmatism.

However, the precise etiology of childhood astigmatism remains elusive. The research conducted on American children has pinpointed several risk factors for astigmatism, which include younger age, Hispanic background, African American race, significant refractive errors such as myopia or hyperopia, and maternal smoking during pregnancy [26, 27]. Additionally, Tong et al. further elucidated that both the corneal curvature radius and AL asymmetry were associated with anisotropic astigmatism [28]. These investigations predominantly centered on anisometropic astigmatism cases. However, there has been a scarcity of studies examining the disparities in ocular biometric parameters between eyes with high astigmatism and those with low astigmatism. During childhood, refractive astigmatism (RA) has been found to be positively correlated with corneal astigmatism (CA). As previously discussed, the development of astigmatism is influenced by the pressure exerted by the eyelids on the eyeball. We hypothesize that ocular biological features, such as corneal thickness and AL, may impact the degree of astigmatism by altering the conformation of the eyeball.

In this study, a nested case–control analysis was conducted, categorizing 240 study eyes into three distinct groups based on astigmatism severity: mild, moderate, and high astigmatism groups. Our analysis revealed a notable finding: Both RA and CA exhibit a negative correlation with CCT. This observation might be attributed to the biomechanical properties of the cornea, where a thinner cornea implies decreased eyeball rigidity. The biomechanical characteristics of the cornea are positively associated with its thickness; thus, under constant intraocular pressure, thinner corneas demonstrate greater compliance to eyelid pressure, potentially resulting in elevated astigmatism formation [29].

Notably, an inverse relationship was observed between the degree of astigmatism and the AL of the eye, and the AL/CR findings echoed the Axl results. The data indicate that as the absolute value of astigmatism diminishes, the corresponding AL increases. This trend could be underpinned by a developmental shift from mixed astigmatism to myopic astigmatism. In cases of myopic astigmatism, one or both meridians focus in front of the retina, introducing a hyperopic blur on the retina that influences AL elongation. This study found that an increase in astigmatism degree was linked to a reduction in myopia levels. This is in line with previous research by Shih et al., which suggested a possible negative correlation between mixed astigmatism and the progression of myopia [30]. Additional research supports our results, indicating that AL growth may alter the anterior segment's structure via eyeball elongation, as evidenced by reduced corneal curvature [31]. This compensatory adjustment in CA predominantly occurs during infancy and early childhood for RA, and it markedly diminishes by the time children reach school age [32]. However, our findings contrast with Li's Nanjing Eye Study [33], which linked AL/CR progression in young children to CA. Discrepancies likely stem from age differences: Li's preschool cohort highlighted corneal-driven astigmatism, while our study may involve older participants where AL effects dominate. Longitudinal studies tracking AL/CR and corneal topography across ages, alongside standardized astigmatism subtypes and multifactorial analyses, are needed to reconcile these observations and inform age-specific interventions.

As for the lens, a critical refractive component closely tied to the eye's focusing mechanism, may profoundly influence myopia progression. Research indicates that once eye axis development stabilizes, LT tends to increase with age [34]. Peng's research on children aged 7 to 15 discovered a lamina LT of 3.45 mm and noted that LT was lesser in low myopia compared to moderate myopia [35]. Shih et al. [30], using Ascan ultrasound, measured the LT of Taiwanese students aged 7 to 18, discovering a gradual decrease in LT from ages 7 to 11, which then increased with age after 12 years. At 18 years, the LT was noted to be the highest, and importantly, the myopic group exhibited the smallest LT value when compared to emmetropic and hyperopic groups. Our research echoes previous findings, revealing a positive correlation between LT and astigmatism degree, while exhibiting a negative correlation with myopia degree.

A striking observation emerged: significant disparities in corneal WTW across the three groups; however, a clear trend was not evident. The nonlinear WTW variation across severity groups implies a complex interaction between corneal anatomy and astigmatism progression. Further longitudinal studies should investigate whether WTW expansion precedes or results from astigmatic changes.

Despite employing a stratified sampling approach, this study has limitations, including the absence of cycloplegic refraction (potentially underestimating astigmatism in hyperopic children), regional/sample size constraints (limiting generalizability), cross-sectional design (precluding longitudinal assessment), interinstrument variability, and unmeasured confounders (e.g., genetics, screen time, and parental myopia). A further critical limitation is the lack of vector-based analysis, which could clarify axis-oculobiometric relationships. While we observed consistent alignment in RA and CA magnitude/axis orientation—suggesting a corneal origin—future studies must incorporate vector decomposition to fully elucidate astigmatism's anisotropic effects. Priorities for future research include large, multicenter longitudinal designs with cycloplegic refraction to track progression, alongside investigations into eyelid anatomy, corneal biomechanics, and region-specific screening guidelines to optimize early detection and management, thereby enhancing visual and academic outcomes in children.

## 5. Conclusion

This study identifies a high prevalence of astigmatism (47.3%) in children in Linzi District, Zibo, exceeding rates in other regions. Contributing factors may include socioeconomic status, early academic pressures, limited outdoor activities, and regional disparities. With-the-rule astigmatism predominates, with severity ≥ 1.00 D significantly impairing VA. Ocular biometric analysis revealed inverse associations between astigmatism severity and CCT or AL, suggesting thinner corneas and shorter AL may predispose to higher astigmatism. Given the risk of amblyopia in severe cases (≤ −2.00 D), early screening is critical. We recommend 1.25 D as a referral threshold for abnormal astigmatism in refractive screenings. Further studies are needed to clarify causal mechanisms and explore eyelid pressure, corneal biomechanics, and LT in astigmatism progression.

## Figures and Tables

**Table 1:** Demographic and astigmatism characteristics of the surveyed students.

Category	Details	Count/percentage
Total participants	N	25,140

Age distribution	7 years	3860 (15.35%)
	8 years	6330 (25.18%)
	9 years	4900 (19.49%)
	10 years	4230 (16.83%)
	11 years	4080 (16.23%)
	12 years	1720 (6.84)
	13 years	20 (0.08%)

Grade categorization	Lower grades (7–10 years)	5820 (23.16%)
	Upper grades (11–13 years)	19,320 (76.84%)

Gender distribution	Male (total sample)	14,014 (55.74%)
	Female (total sample)	11,126 (44.26%)

Astigmatism prevalence	Total astigmatism	11,616 (46.21)
	Mild astigmatism	9341 (37.16%)
	Moderate astigmatism	1712 (6.81%)
	High astigmatism	563 (2.24%)

Gender in astigmatism	Male	6520 (56.13%)
	Female	5096 (43.87%)

**Table 2:** Comparison of lower and upper grades.

	Lower grades	Upper grades	F value	*p* value
Visual acuity (LogMAR)	0.09 ± 0.178	0.23 ± 0.289	14.92	< 0.001
Spherical diopter (D)	−0.26 ± 1.190	−0.94 ± 1.723	11.68	< 0.001
Cylinder diopter (D)	−0.33 ± 0.647	−0.50 ± 0.766	5.87	< 0.001
Axis position	81.68 ± 67.259	88.11 ± 66.781	−2.38	0.02

*Note:* D: diopter.

**Table 3:** Comparison of different degrees of astigmatism among 11,616 students.

	Astigmatism			F value	*p* value
	Mild	Moderate	High		
Age (years)	9.35 ± 1.543	9.20 ± 1.641	9.67 ± 1.552	1.91	0.15
Visual acuity (LogMAR)	0.18 ± 0.256	0.24 ± 0.287	0.29 ± 0.280	8.94	< 0.001
Spherical diopter (D)	−0.78 ± 1.445	−0.58 ± 1.886	−0.35 ± 2.171	3.00	0.04

*Note:* D: diopter.

**Table 4:** Comparative analysis of biological parameters across varying degrees of astigmatism in 240 children.

	Astigmatism			F/Chi-square value	*p* value
	Mild (*n* = 96)	Moderate (*n* = 80)	High (*n* = 64)		
Age (y)	8.41 ± 1.798	8.41 ± 1.223	8.34 ± 2.421	3.83	0.07
Gender (M/F)	54/42	51/29	46/18	2.35	0.31
RA (D)	−0.77 ± 0.214	−1.51 ± 0.254	−3.01 ± 0.627	672.87	< 0.001
Axis1	85.45 ± 84.085	83.80 ± 85.032	102.83 ± 84.776	1.082	0.34
CA (D)	−0.75 ± 0.322	−1.55 ± 0.345	−3.12 ± 0.721	498.25	< 0.001
Axis2	84.72 ± 81.896	87.68 ± 83.490	102.17 ± 83.139	0.92	0.40
CCT (μm)	540.29 ± 24.221	536.64 ± 26.815	528.97 ± 31.544	3.69	0.03
Axl (mm)	24.06 ± 0.888	23.66 ± 1.376	22.77 ± 1.189	24.38	< 0.001
K1 (D)	42.60 ± 1.648	42.15 ± 1.816	42.73 ± 2.111	2.12	0.12
K2 (D)	43.36 ± 1.616	43.68 ± 1.860	45.84 ± 2.155	37.92	< 0.001
LT (mm)	3.40 ± 0.189	3.40 ± 0.215	3.50 ± 0.201	6.75	< 0.001
WTW (mm)	12.12 ± 0.346	12.30 ± 0.417	12.20 ± 0.518	3.59	0.03
Km (D)	42.98 ± 1.624	42.92 ± 1.828	44.28 ± 2.104	12.54	< 0.001
A/K ratio	3.06 ± 0.117	3.01 ± 0.177	2.98 ± 0.540	5.95	0.01

*Note:* D: diopter; Axis1: the axis of refractive astigmatism; Axis2: the axis of corneal astigmatism; Axl: axial length; K1: flat keratometry; K2: steep keratometry; WTW: horizontal white-to-white; Km: mean keratometry; A/K ratio: the ratio of axis length and keratometry.

Abbreviations: CA, corneal astigmatism; CCT, central corneal thickness; LT, lens thickness; RA, refractive astigmatism.

## Data Availability

The datasets presented in this study are all included within this article, further inquiries can be available from the corresponding author.
